# Supplementary material for: Effects of defoliation and nitrogen on carbon dioxide (CO2) emissions and microbial communities in soils of cherry tree orchards
Source: PeerJ. 2023 May 8;11:e15276. doi: 10.7717/peerj.15276 (PMC10174058; doi:10.7717/peerj.15276)
Supplement: Supplementary file 1 [file peerj-11-15276-s001.docx]

ANOVA

				Sum of Squares	df	Mean Square	F	Sig.
TMBC4	Between Groups	(Combined)		287997.5	5	57599.490	74.571	.000
		Linear Term	Contrast	35902.512	1	35902.512	46.481	.000
			Deviation	252094.9	4	63023.735	81.593	.000
	Within Groups			9268.941	12	772.412		
	Total			297266.4	17			
TMBC13	Between Groups	(Combined)		40405.152	5	8081.030	10.747	.000
		Linear Term	Contrast	1681.416	1	1681.416	2.236	.161
			Deviation	38723.735	4	9680.934	12.874	.000
	Within Groups			9023.366	12	751.947		
	Total			49428.518	17			
TMBC80	Between Groups	(Combined)		22601.288	5	4520.258	14.972	.000
		Linear Term	Contrast	610.850	1	610.850	2.023	.180
			Deviation	21990.438	4	5497.609	18.209	.000
	Within Groups			3622.952	12	301.913		
	Total			26224.240	17			
MMBC4	Between Groups	(Combined)		233103.4	5	46620.686	57.545	.000
		Linear Term	Contrast	14834.237	1	14834.237	18.310	.001
			Deviation	218269.2	4	54567.299	67.354	.000
	Within Groups			9721.850	12	810.154		
	Total			242825.3	17			
MMBC13	Between Groups	(Combined)		145976.9	5	29195.376	76.424	.000
		Linear Term	Contrast	9214.514	1	9214.514	24.121	.000
			Deviation	136762.4	4	34190.591	89.500	.000
	Within Groups			4584.201	12	382.017		
	Total			150561.1	17			
MMBC80	Between Groups	(Combined)		10513.503	5	2102.701	10.569	.000
		Linear Term	Contrast	2364.638	1	2364.638	11.886	.005
			Deviation	8148.865	4	2037.216	10.240	.001
	Within Groups			2387.407	12	198.951		
	Total			12900.910	17			
DMBC4	Between Groups	(Combined)		14070.009	5	2814.002	6.945	.003
		Linear Term	Contrast	57.703	1	57.703	.142	.712
			Deviation	14012.306	4	3503.077	8.645	.002
	Within Groups			4862.403	12	405.200		
	Total			18932.412	17			
DMBC13	Between Groups	(Combined)		16224.509	5	3244.902	7.707	.002
		Linear Term	Contrast	3730.149	1	3730.149	8.860	.012
			Deviation	12494.360	4	3123.590	7.419	.003
	Within Groups			5052.169	12	421.014		
	Total			21276.677	17			
DMBC80	Between Groups	(Combined)		22154.766	5	4430.953	15.354	.000
		Linear Term	Contrast	751.124	1	751.124	2.603	.133
			Deviation	21403.642	4	5350.910	18.542	.000
	Within Groups			3463.035	12	288.586		
	Total			25617.801	17			
TCatalase	Between Groups	(Combined)		467.711	5	93.542	97.218	.000
		Linear Term	Contrast	205.861	1	205.861	213.950	.000
			Deviation	261.850	4	65.462	68.035	.000
	Within Groups			11.546	12	.962		
	Total			479.257	17			
MCatalase	Between Groups	(Combined)		328.554	5	65.711	165.560	.000
		Linear Term	Contrast	206.951	1	206.951	521.419	.000
			Deviation	121.603	4	30.401	76.595	.000
	Within Groups			4.763	12	.397		
	Total			333.316	17			
DCatalase	Between Groups	(Combined)		103.013	5	20.603	58.113	.000
		Linear Term	Contrast	4.138	1	4.138	11.673	.005
			Deviation	98.874	4	24.719	69.723	.000
	Within Groups			4.254	12	.355		
	Total			107.267	17			
Talkaline	Between Groups	(Combined)		1046.536	5	209.307	65.472	.000
		Linear Term	Contrast	301.266	1	301.266	94.238	.000
			Deviation	745.269	4	186.317	58.281	.000
	Within Groups			38.363	12	3.197		
	Total			1084.898	17			
Malkaline	Between Groups	(Combined)		335.898	5	67.180	70.171	.000
		Linear Term	Contrast	89.168	1	89.168	93.138	.000
			Deviation	246.730	4	61.683	64.429	.000
	Within Groups			11.488	12	.957		
	Total			347.386	17			
Dalkaline	Between Groups	(Combined)		57.073	5	11.415	25.514	.000
		Linear Term	Contrast	11.160	1	11.160	24.944	.000
			Deviation	45.913	4	11.478	25.656	.000
	Within Groups			5.369	12	.447		
	Total			62.442	17			
Tcellulase	Between Groups	(Combined)		9.432	5	1.886	125.478	.000
		Linear Term	Contrast	2.035	1	2.035	135.334	.000
			Deviation	7.397	4	1.849	123.014	.000
	Within Groups			.180	12	.015		
	Total			9.612	17			
Mcellulase	Between Groups	(Combined)		7.595	5	1.519	225.954	.000
		Linear Term	Contrast	2.116	1	2.116	314.781	.000
			Deviation	5.479	4	1.370	203.748	.000
	Within Groups			.081	12	.007		
	Total			7.675	17			
Dcellulase	Between Groups	(Combined)		4.090	5	.818	141.713	.000
		Linear Term	Contrast	1.076	1	1.076	186.361	.000
			Deviation	3.014	4	.754	130.550	.000
	Within Groups			.069	12	.006		
	Total			4.159	17			

TMBC4

	´¦Àí	N	Subset for alpha = .05		
			1	2	3
Tukey B[a]	3.00	3	185.3600		
	5.00	3	217.2600		
	1.00	3	251.6500		
	2.00	3		419.5567	
	4.00	3		473.3067	473.3067
	6.00	3			498.4933

Means for groups in homogeneous subsets are displayed.

a. Uses Harmonic Mean Sample Size = 3.000.

TMBC13

	´¦Àí	N	Subset for alpha = .05	
			1	2
Tukey B[a]	1.00	3	278.6300	
	5.00	3	298.2133	
	6.00	3	331.6933	
	3.00	3	337.0067	
	2.00	3	351.0533	
	4.00	3		428.2833

Means for groups in homogeneous subsets are displayed.

a. Uses Harmonic Mean Sample Size = 3.000.

TMBC80

	´¦Àí	N	Subset for alpha = .05		
			1	2	3
Tukey B[a]	5.00	3	182.5500		
	2.00	3	224.6700	224.6700	
	3.00	3	224.6700	224.6700	
	1.00	3		231.6933	
	6.00	3		266.8000	266.8000
	4.00	3			294.8833

Means for groups in homogeneous subsets are displayed.

a. Uses Harmonic Mean Sample Size = 3.000.

MMBC4

	´¦Àí	N	Subset for alpha = .05		
			1	2	3
Tukey B[a]	5.00	3	136.7333		
	3.00	3	168.5067	168.5067	
	1.00	3		221.7233	
	2.00	3			366.8000
	4.00	3			379.1367
	6.00	3			435.3033

Means for groups in homogeneous subsets are displayed.

a. Uses Harmonic Mean Sample Size = 3.000.

MMBC13

	´¦Àí	N	Subset for alpha = .05		
			1	2	3
Tukey B[a]	5.00	3	140.4200		
	3.00	3	172.0200	172.0200	
	1.00	3		196.5900	
	2.00	3			322.9667
	4.00	3			349.3467
	6.00	3			363.3900

Means for groups in homogeneous subsets are displayed.

a. Uses Harmonic Mean Sample Size = 3.000.

MMBC80

	´¦Àí	N	Subset for alpha = .05	
			1	2
Tukey B[a]	3.00	3	168.5100	
	5.00	3	175.5300	
	1.00	3	182.5500	
	2.00	3	188.4933	
	6.00	3		224.6700
	4.00	3		231.6933

Means for groups in homogeneous subsets are displayed.

a. Uses Harmonic Mean Sample Size = 3.000.

DMBC4

	´¦Àí	N	Subset for alpha = .05	
			1	2
Tukey B[a]	3.00	3	105.3167	
	5.00	3	112.9033	
	1.00	3	118.6167	
	6.00	3	119.3567	
	2.00	3	129.9900	
	4.00	3		189.5700

Means for groups in homogeneous subsets are displayed.

a. Uses Harmonic Mean Sample Size = 3.000.

DMBC13

	´¦Àí	N	Subset for alpha = .05		
			1	2	3
Tukey B[a]	3.00	3	143.8067		
	1.00	3	168.5067	168.5067	
	6.00	3		205.5267	205.5267
	4.00	3		208.0467	208.0467
	2.00	3		216.4667	216.4667
	5.00	3			231.6933

Means for groups in homogeneous subsets are displayed.

a. Uses Harmonic Mean Sample Size = 3.000.

DMBC80

	´¦Àí	N	Subset for alpha = .05	
			1	2
Tukey B[a]	4.00	3	161.4867	
	5.00	3	168.1767	
	3.00	3	168.5067	
	1.00	3	175.5300	
	2.00	3		238.7133
	6.00	3		245.7333

Means for groups in homogeneous subsets are displayed.

a. Uses Harmonic Mean Sample Size = 3.000.

TCatalase

	´¦Àí	N	Subset for alpha = .05			
			1	2	3	4
Tukey B[a]	3.00	3	14.4690			
	2.00	3	15.5190			
	5.00	3		19.5090		
	1.00	3		20.0590		
	4.00	3			23.2890	
	6.00	3				29.7623

Means for groups in homogeneous subsets are displayed.

a. Uses Harmonic Mean Sample Size = 3.000.

MCatalase

	´¦Àí	N	Subset for alpha = .05			
			1	2	3	4
Tukey B[a]	2.00	3	15.0990			
	3.00	3	15.0990			
	1.00	3		18.7990		
	5.00	3			22.0290	
	4.00	3			22.4490	
	6.00	3				27.0690

Means for groups in homogeneous subsets are displayed.

a. Uses Harmonic Mean Sample Size = 3.000.

DCatalase

	´¦Àí	N	Subset for alpha = .05		
			1	2	3
Tukey B[a]	1.00	3	2.7090		
	5.00	3		5.5990	
	6.00	3		6.7623	
	3.00	3			8.9040
	2.00	3			9.2190
	4.00	3			9.3240

Means for groups in homogeneous subsets are displayed.

a. Uses Harmonic Mean Sample Size = 3.000.

Talkaline

	´¦Àí	N	Subset for alpha = .05		
			1	2	3
Tukey B[a]	3.00	3	15.1655		
	1.00	3	15.3000		
	5.00	3		21.7260	
	2.00	3			29.6667
	4.00	3			32.5133
	6.00	3			33.3633

Means for groups in homogeneous subsets are displayed.

a. Uses Harmonic Mean Sample Size = 3.000.

Malkaline

	´¦Àí	N	Subset for alpha = .05			
			1	2	3	4
Tukey B[a]	3.00	3	9.0533			
	1.00	3		12.9333		
	5.00	3		13.9433		
	2.00	3			17.8067	
	4.00	3			17.9567	
	6.00	3				22.5933

Means for groups in homogeneous subsets are displayed.

a. Uses Harmonic Mean Sample Size = 3.000.

Dalkaline

	´¦Àí	N	Subset for alpha = .05		
			1	2	3
Tukey B[a]	1.00	3	5.0533		
	2.00	3	5.9367		
	5.00	3	5.9900		
	6.00	3		7.9000	
	3.00	3		8.4933	
	4.00	3			10.2367

Means for groups in homogeneous subsets are displayed.

a. Uses Harmonic Mean Sample Size = 3.000.

Tcellulase

	´¦Àí	N	Subset for alpha = .05			
			1	2	3	4
Tukey B[a]	3.00	3	.8367			
	5.00	3	.8600			
	1.00	3	.9200			
	2.00	3		1.8867		
	4.00	3			2.2400	
	6.00	3				2.6333

Means for groups in homogeneous subsets are displayed.

a. Uses Harmonic Mean Sample Size = 3.000.

Mcellulase

	´¦Àí	N	Subset for alpha = .05		
			1	2	3
Tukey B[a]	1.00	3	.6533		
	5.00	3	.6567		
	3.00	3	.6867		
	2.00	3		1.5267	
	4.00	3		1.5400	
	6.00	3			2.4100

Means for groups in homogeneous subsets are displayed.

a. Uses Harmonic Mean Sample Size = 3.000.

Dcellulase

	´¦Àí	N	Subset for alpha = .05			
			1	2	3	4
Tukey B[a]	3.00	3	.3700			
	1.00	3	.3867			
	5.00	3	.4133			
	2.00	3		.9867		
	4.00	3			1.3333	
	6.00	3				1.5400

Means for groups in homogeneous subsets are displayed.

a. Uses Harmonic Mean Sample Size = 3.000.

Tests of Between-Subjects Effects

Source	Dependent Variable	Type III Sum of Squares	df	Mean Square	F	Sig.
Corrected Model	Proteobacteria	.177[a]	17	.010	26.274	.000
	Acidobacteria	.073[b]	17	.004	28.859	.000
	CO2ef	8444.593[c]	17	496.741	96.316	.000
	cumCO2	4.309[d]	17	.253	654.945	.000
	MBC	967820.190[e]	17	56930.599	85.921	.000
	catalase	2814.913[f]	17	165.583	265.051	.000
	alkaline	4150.639[g]	17	244.155	159.175	.000
	celluloytic	25.864[g]	17	1.521	165.805	.000
Intercept	Proteobacteria	12.547	1	12.547	31661.724	.000
	Acidobacteria	2.120	1	2.120	14311.250	.000
	CO2ef	33202.241	1	33202.241	6437.777	.000
	cumCO2	30.091	1	30.091	77746.225	.000
	MBC	3419520.862	1	3419520.862	5160.850	.000
	catalase	13824.192	1	13824.192	22128.542	.000
	alkaline	13597.553	1	13597.553	8864.818	.000
	celluloytic	79.789	1	79.789	8695.479	.000
s	Proteobacteria	.055	2	.027	68.921	.000
	Acidobacteria	.019	2	.009	63.838	.000
	CO2ef	1149.731	2	574.866	111.464	.000
	cumCO2	.661	2	.331	854.388	.000
	MBC	432649.299	2	216324.649	326.484	.000
	catalase	1963.306	2	981.653	1571.343	.000
	alkaline	2711.132	2	1355.566	883.751	.000
	celluloytic	4.748	2	2.374	258.703	.000
n	Proteobacteria	.009	2	.005	11.808	.000
	Acidobacteria	.007	2	.004	25.288	.000
	CO2ef	470.065	2	235.032	45.572	.000
	cumCO2	.076	2	.038	97.699	.000
	MBC	15724.741	2	7862.370	11.866	.000
	catalase	63.518	2	31.759	50.837	.000
	alkaline	1.673	2	.837	.545	.584
	celluloytic	1.188	2	.594	64.753	.000
c	Proteobacteria	.027	1	.027	68.416	.000
	Acidobacteria	.001	1	.001	4.050	.052
	CO2ef	4356.019	1	4356.019	844.614	.000
	cumCO2	3.193	1	3.193	8248.656	.000
	MBC	371728.832	1	371728.832	561.025	.000
	catalase	140.006	1	140.006	224.109	.000
	alkaline	824.039	1	824.039	537.225	.000
	celluloytic	17.727	1	17.727	1931.955	.000
s * n	Proteobacteria	.014	4	.004	9.047	.000
	Acidobacteria	.005	4	.001	9.238	.000
	CO2ef	518.380	4	129.595	25.128	.000
	cumCO2	.022	4	.005	13.928	.000
	MBC	5663.684	4	1415.921	2.137	.096
	catalase	36.752	4	9.188	14.707	.000
	alkaline	25.991	4	6.498	4.236	.007
	celluloytic	.231	4	.058	6.298	.001
s * c	Proteobacteria	.019	2	.009	23.584	.000
	Acidobacteria	.004	2	.002	15.113	.000
	CO2ef	373.787	2	186.894	36.238	.000
	cumCO2	.022	2	.011	28.368	.000
	MBC	119168.687	2	59584.343	89.927	.000
	catalase	19.950	2	9.975	15.967	.000
	alkaline	377.423	2	188.712	123.029	.000
	celluloytic	.529	2	.265	28.846	.000
n * c	Proteobacteria	.045	2	.023	56.836	.000
	Acidobacteria	.018	2	.009	59.663	.000
	CO2ef	364.287	2	182.144	35.317	.000
	cumCO2	.296	2	.148	382.139	.000
	MBC	6934.500	2	3467.250	5.233	.010
	catalase	264.480	2	132.240	211.678	.000
	alkaline	105.920	2	52.960	34.527	.000
	celluloytic	1.293	2	.646	70.444	.000
s * n * c	Proteobacteria	.008	4	.002	4.939	.003
	Acidobacteria	.018	4	.005	30.450	.000
	CO2ef	1212.324	4	303.081	58.766	.000
	cumCO2	.040	4	.010	26.129	.000

Multiple Comparisons

LSD

Dependent Variable	(I) soildepth	(J) soildepth	Mean Difference (I-J)	Std. Error	Sig.	95% Confidence Interval	
						Lower Bound	Upper Bound
chao1	1	2	308.9969*	78.63463	.000	149.5184	468.4753
		3	394.3364*	78.63463	.000	234.8580	553.8148
	2	1	-308.9969*	78.63463	.000	-468.4753	-149.5184
		3	85.3395	78.63463	.285	-74.1389	244.8179
	3	1	-394.3364*	78.63463	.000	-553.8148	-234.8580
		2	-85.3395	78.63463	.285	-244.8179	74.1389
shannon	1	2	.4171	.21260	.058	-.0141	.8483
		3	.6362*	.21260	.005	.2050	1.0674
	2	1	-.4171	.21260	.058	-.8483	.0141
		3	.2191	.21260	.310	-.2121	.6503
	3	1	-.6362*	.21260	.005	-1.0674	-.2050
		2	-.2191	.21260	.310	-.6503	.2121
simpson	1	2	.0100	.00939	.296	-.0091	.0290
		3	.0126	.00939	.188	-.0064	.0317
	2	1	-.0100	.00939	.296	-.0290	.0091
		3	.0026	.00939	.780	-.0164	.0217
	3	1	-.0126	.00939	.188	-.0317	.0064
		2	-.0026	.00939	.780	-.0217	.0164

Based on observed means.

*. The mean difference is significant at the .05 level.
